# Hybrid CNN-GRU Models for Improved EEG Motor Imagery Classification

**DOI:** 10.3390/s25051399

**Published:** 2025-02-25

**Authors:** Mouna Bouchane, Wei Guo, Shuojin Yang

**Affiliations:** 1 Key Laboratory of Augmented Reality, School of Mathematical Sciences, Hebei Normal University, Shijiazhuang 050024, China; mouna.bouchane@gmail.com; 2 Department of Computer Science and Technology, Tsinghua University, Beijing 100084, China; yangshuojin@tsinghua.edu.cn

**Keywords:** convolutional neural network (CNN), gated recurrent unit (GRU), bidirectional GRU, motor imagery (MI), electroencephalography (EEG), hybrid models

## Abstract

Brain–computer interfaces (BCIs) based on electroencephalography (EEG) enable neural activity interpretation for device control, with motor imagery (MI) serving as a key paradigm for decoding imagined movements. Efficient feature extraction from raw EEG signals is essential to improve classification accuracy while minimizing reliance on extensive preprocessing. In this study, we introduce new hybrid architectures to enhance MI classification using data augmentation and a limited number of EEG channels. The first model combines a shallow convolutional neural network and a gated recurrent unit (CNN-GRU), while the second incorporates a convolutional neural network with a bidirectional gated recurrent unit (CNN-Bi-GRU). Evaluated using the publicly available PhysioNet dataset, the CNN-GRU classifier achieved peak mean accuracy rates of 99.71%, 99.73%, 99.61%, and 99.86% for tasks involving left fist (LF), right fist (RF), both fists (LRF), and both feet (BF), respectively. The experimental results provide compelling evidence that our proposed models outperform current state-of-the-art methods, underscoring their efficiency on small-scale EEG datasets. The CNN-GRU and CNN-Bi-GRU architectures exhibit superior predictive reliability, offering a faster, cost-effective solution for user-adaptable MI-BCI applications.

## 1. Introduction

Brain–computer interfaces (BCIs) facilitate direct communication between the brain and external devices, with applications spanning from assisting individuals with cognitive or motor impairments to neurorehabilitation and augmentative communication systems [1,2]. The effectiveness of BCIs depends on advanced signal processing and classification methods, particularly in decoding motor imagery (MI) tasks from neural signals.

Motor imagery is the mental simulation of movement without physical execution, engaging brain regions associated with actual motion. In brain–computer interfaces (BCIs), these neural signals, recorded via electroencephalography (EEG), are decoded to enable users to control external devices through thought, providing a non-invasive neural interaction framework. Although EEG-based MI-BCI systems hold considerable promise for assistive technology and rehabilitation, their reliability and practical application are hindered by several constraints [1]. One of these challenges is low signal-to-noise ratio (SNR), mainly due to artifacts from muscle activity, eye movement, and other external sources, which complicates the accurate interpretation of neural signals. Moreover, the limited availability of labeled data and the inherently non-stationary nature of brain signals further compromise decoding accuracy [3,4].

To address these issues, researchers have focused on refining feature extraction, and classification to capture relevant neural patterns while minimizing extraneous noise. Various techniques have been developed for MI task recognition, each offering distinct advantages. Common spatial pattern (CSP) effectively extracts EEG features for two classes by focusing on event-related desynchronization/synchronization (ERD/ERS) [5,6]. Linear discriminant analysis (LDA) and support vector machines (SVM) are also commonly applied for their accurate classification [7,8].

In recent years, deep learning approaches, especially convolutional neural networks (CNNs), excel in capturing spatial dependencies in EEG data [9], whereas recurrent neural networks (RNNs) including long short-term memory (LSTM) and gated recurrent units (GRUs), are noted for their ability to model temporal dependencies [10,11]. Recent works have emphasized improving temporal modeling to decode brain EEG signals into controlled digital commands [12]. Asghar et al. introduced a semi-skipping layer for GRUs in RNNs, showcasing their effectiveness in capturing temporal dependencies for edge computing in EEG-based emotion classification [13]. In [14], Songram et al. developed a combined CNN with long short-term memory (LSTM) layers for medical image classification, underlining the potential of hybrid design compared to other architectures. Moreover, Xu et al. explored the synergistic integration of CNN and RNN components within a single framework for various classification tasks, to extract temporal frequency and spatial features [15]. The information hidden in the middle filters of a neural network can extract low-level features, leading to a drastic decrease in the number of required parameters and enhancing computational efficiency.

In this study, we propose two novel hybrid architectures: CNN-GRU and CNN-Bi-GRU for robust classification of four MI tasks, including a baseline as a fifth class to improve system stability and control accuracy. The methodology follows a structured preprocessing pipeline. Optimal EEG channel selection reduces data dimensionality while preserving essential discriminative features. Data filtering and normalization mitigate artifacts and standardize input distributions, ensuring cleaner and more interpretable signals. In addition, we employ the synthetic minority oversampling technique (SMOTE) to mitigate class imbalance and the scarcity of labeled data, thereby enhancing model generalization and expediting training. Feature extraction and classification are performed using CNN-GRU and CNN-Bi-GRU architectures. The CNN layers effectively capture spatial dependencies in EEG signals, while GRUs model temporal dynamics, resulting in enhanced MI task discrimination. Our approach was evaluated using the publicly available PhysioNet dataset [16], achieving an outstanding accuracy of 99.65%, surpassing state-of-the-art models. These results underscore the efficiency, adaptability, and suitability for low-cost applicability of our hybrid architectures, reinforcing their potential for practical BCI implementations in neurorehabilitation and assistive technologies. The main contributions of this paper are summarized as follows:

(a) Novel Hybrid Architectures: We combine shallow CNN, GRU, and Bi-GRU to design two architectures that effectively preserve spatial and temporal feature extraction and improve the decoding robustness of the five-class motor imagery data.
(b) SMOTE Data Augmentation: The use of SMOTE addresses class imbalance, artificially increases the size of the training dataset, tackles overfitting, and boosts model generalization.
(c) Improved Performance and Broad Applicability: Various performance metrics achieve superior accuracy relative to conventional methods and underscore the reliability of our approach using limited channel, small-size data for BCI MI systems, offering a cost-effective solution for future neurorehabilitation and assistive technologies applications.

## 2. Related Work

### 2.1. Data Augmentation

Class imbalance is a prevalent obstacle in motor imagery (MI) classification due to the uneven distribution of trials across different classes in EEG datasets. To address this issue, researchers have increasingly adopted data augmentation techniques, such as the synthetic minority oversampling technique (SMOTE). SMOTE has gained attention for its ability to generate synthetic samples of the minority class; SMOTE enhances the representation of underrepresented classes, which can lead to improved classifier performance. Lee et al. discussed the effects of using borderline-SMOTE for data augmentation in P300-based brain–computer interfaces classification tasks, highlighting its relevance to BCI systems [17]. In similar vein, Bej et al. evaluated various oversampling techniques, including SMOTE, for addressing class imbalance in datasets and demonstrated its effectiveness in enhancing recognition [18]. Furthermore, Wang et al. proposed a SMOTE algorithm based on normal distribution, which generates synthetic samples closer to the class center. This approach not only ensures better alignment with original data characteristics but also improves classification accuracy, particularly with random forest models [19]. The synthetic samples created by SMOTE allow classifiers to learn more generalized patterns, leading to more reliable predictions on unseen data. Mustaqim et al. [20] compared SMOTE with other oversampling and undersampling methods, showing its superiority in managing class imbalance and alleviating the overfitting problem. Figueiredo et al. validated SMOTE’s applicability in real-time brain–computer interface (BCI) systems, showing its ability to maintain model stability and reduce bias during live MI classification tasks [21]. SMOTE contributed to improving the training process of neural networks that tend to suffer from poor robustness, particularly in scenarios with limited data. In biomedical contexts, where it is very difficult to collect large-scale data, the findings indicated that combining SMOTE with neural networks led to improved accuracy performance [22]. However, the effectiveness of SMOTE can vary based on the dimensionality of the data and the specific classifiers employed, making it essential to consider these factors carefully during application [23]. The method’s performance is strongly influenced by dataset characteristics and classifier selection, reinforcing the need for rigorous preprocessing steps for a well-prepared dataset and appropriate classifier choice for optimal results.

### 2.2. Gated Recurrent Unit

BCIs bridge the human–computer gap by translating brain signals into intentional commands, to create an efficient, high-accuracy communication link between brain processing and external devices. Previous research focused on developing a motor imagery-based brain–computer interface (MI-BCI) utilizing recurrent neural networks to enable direct and intuitive control of computer applications through brain activity. However, traditional RNNs often struggle with long-term dependencies due to issues such as vanishing and exploding gradients [24].

Gated recurrent units (GRUs) are particularly effective for sequence classification tasks such as those required in BCIs [25]. Their gating mechanisms, consisting of reset and update gates, enable GRUs to manage long-term dependencies in data by selectively controlling the flow of information. The reset gate determines how much past information to discard, while the update gate decides how much new information to incorporate and carry forward, ensuring effective temporal pattern learning [26].

GRUs excel at capturing the intricate temporal dynamics of EEG signals, facilitating accurate classification of motor imagery states. This capability enhances the robustness and reliability of brain–computer interfaces, establishing GRU-based methods as potent tools for decoding complex neural activity [27].

GRU classification strategies show significant potential for MI-BCI systems due to their capacity to handle successive information and capture long-term conditions. Compared to LSTM, GRUs are more computationally efficient, requiring fewer tensor operations and enabling faster training. Despite these advantages, GRU effectiveness depends on access to substantial labeled datasets and robust strategies to prevent overfitting, such as careful validation and regularization [28].

### 2.3. Convolutional Neural Network for BCI Systems

The domain of motor imagery-based brain–computer interfaces (MI-BCIs) has witnessed substantial evolution, with convolutional neural networks (CNNs) assuming a pivotal position. This chapter explores key research endeavors that demonstrate the versatility and proficiency of shallow CNNs in managing input representations and extraction methods for MI-BCI classification.

Dose et al. [29] implemented a CNN capable of classifying raw signals related to a four-task MI dataset with maximum accuracy of 80.10% using different numbers of EEG channels from 9 to 64. Tang et al. [30] introduced a classification method for a two-task motor imagery (MI) dataset comprising 360 instances and 16 EEG channels, using a combination of CNN and Empirical Mode Decomposition (EMD). This approach reached an average accuracy of 85.83% per participant. More recently, Lun et al. [31] proposed CNN architecture able to classify a four-task MI dataset achieving 95.76% average accuracy on multiple participants, introducing the selection of symmetrical channels pairs situated near the central brain sulcus, as input to the neural network.

In parallel, one seminal work by Sakhavi et al. [32] proposed a modified filter-bank common spatial patterns method, optimizing a shallow CNN that demonstrated efficacy in capturing discriminative features crucial for MI-BCI. Similarly, Kwon et al. [33] displayed the adaptive nature of CNNs in processing diverse inputs for MI-BCI classification by utilizing spectral-spatial inputs from discriminative frequency bands. In the study conducted by Dai et al. [34], a novel input modeling was devised by amalgamating time, frequency, and channel information from EEG signals. The results underscored the potential of shallow CNNs in effectively handling diverse input modalities for MI-BCI classification. Leewis et al. [35] integrated common spatial patterns (CSP) and linear discriminant analysis (LDA) within a shallow CNN framework. This integration exhibited the adaptability of shallow CNNs in synergizing with traditional feature extraction methods for MI-BCI classification. Contributing to this body of knowledge, Zahra et al. [36] employed a CNN approach for processing entire input data, illustrating the versatility of shallow CNNs in seamless data processing and classification for MI-BCI systems.

The findings from diverse studies underscore the crucial role played by shallow convolutional neural network (CNN) architectures in advancing motor imagery-based brain-computer interface (MI-BCI) systems. CNNs have demonstrated impressive adaptability to various input representations and feature extraction methodologies, contributing significantly to the increased efficiency and superior classification performance observed in MI-BCI systems [37]. Acknowledging the trade-off of extended training times, this approach establishes a pathway for swifter online testing and classification during deployment. Noteworthy is the achievement of this efficiency with reduced computational resources and heightened accuracy. Despite the significant strides made in capitalizing on CNN for MI-BCI systems, the current literature in this domain remains relatively sparse. This underscores the compelling need for further exploration of preprocessing techniques and convolutional neural network architectures. These efforts are poised to fully unlock the potential of MI-BCI applications, ushering in a new era of advancements in brain–computer interfaces [38,39,40].

The next section outlines the process of acquiring EEG signals from the Physionet dataset to examine how different channel subsets affect decoding performance and describes the architectures of the proposed learning models. Section 4 presents the results of the analysis and classification, along with a detailed examination of the experimental findings. Section 5 provides a discussion and comparison with state-of-the-art methods. Finally, Section 6 concludes this study.

## 3. Methods

Our proposed approach introduces a hybrid model using a four-layered one-dimensional convolutional neural network (1D-CNN) and a gated recurrent unit (GRU). This model is carefully designed to integrate spatial and temporal features and predict five different mental states induced by motor imagery, including a baseline class. The SMOTE data augmentation technique is employed to address MI EEG data class imbalance.

### 3.1. Dataset Implementation

The dataset utilized in this study was sourced from the EEG Motor Movement/Imagery Dataset V 1.0.0 [16]. It contains EEG recordings from 109 participants engaged in four tasks across 14 experimental runs. Due to annotation errors, data from participants 38, 88, 89, 92, 100, and 104 were excluded, resulting in a dataset of 103 participants. During the motor imagery (MI) tasks, EEG signals were recorded using a 64-channel setup with the BCI2000 system, following the international 10-10 system but excluding electrodes Nz, F9, F10, FT9, FT10, A1, A2, TP9, TP10, P9, and P10. The recordings were sampled at 160 Hz with an average reference. The five MI classes are detailed in Table 1.

The approach employs the class label LF for T1, which corresponds to the imagined movement of the left fist when the task involves a target appearing on either the left or right side of the screen. In this scenario, the participant imagines opening and closing their left fist until the target vanishes, followed by relaxation. The label LRF for T1 is used when the target appears at the top or bottom of the screen; participants imagine moving both fists if the target is on top or both feet if it is at the bottom, relaxing afterward. For T2, the label RF is applied to the imagined movement of the right fist, similar to T1 but with the right fist. When the target appears on the top or bottom, participants again imagine moving both fists or both feet, respectively. The class label BF for T2 is associated with the task involving the movement of both feet. Lastly, a fifth class B represents the baseline state.

### 3.2. Data Subsets

**Figure 1:**
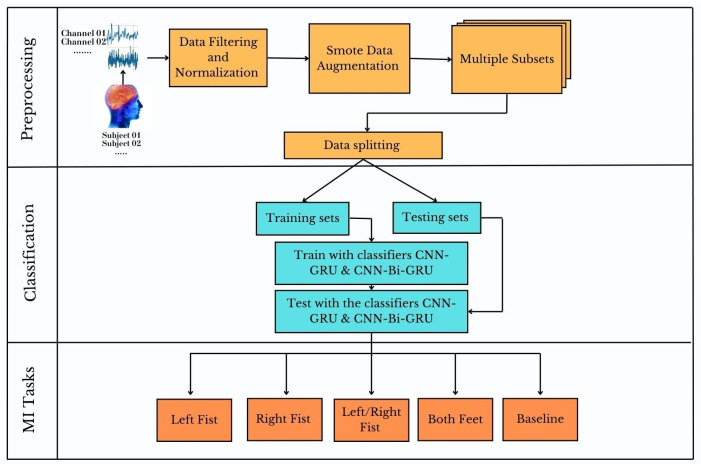
Schematic overview of the proposed framework, including preprocessing of raw signals from selected channels of 7 subjects, followed by the classification of 4 motor imagery (MI) tasks and a baseline class using the convolutional neural network–gated recurrent unit (CNN-GRU) and convolutional neural network-bidirectional gated recurrent unit (CNN-Bi-GRU) models.

**Figure 2:**
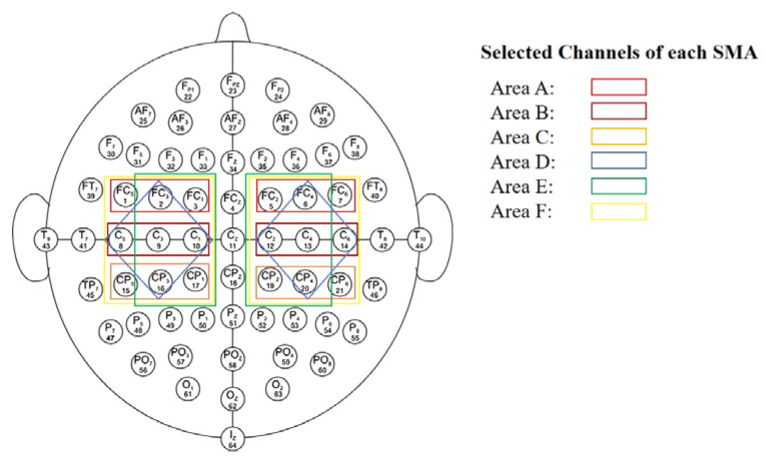
Visualization of the six sensorimotor areas (SMAs) labeled ‘A’ through ‘F’, chosen in proximity of the sensorimotor cortex. With 64 EEG electrode montage, standardized according to the international 10–20 system, each of the six SMAs corresponds to a distinct rectangle, demarking the selected channel pairs combination and using various colors for clarity and ease of interpretation.

In order to have a good base for comparison of the proposed approach and building on the field literature and previous works [31,37,41], we considered the six sensorimotor areas (SMAs) illustrated in Figure 1. Consequently, we created and used six different subsets extracted from the original dataset, covering different SMAs. For each SMA, each experimental run is divided into 4 s segments and is assigned a label corresponding to one of the five classes used (LF, RF, LRF, BF, B). Each instance within the dataset consists of a matrix of size 640 × 2; 2 corresponds to a pair of specular channels in the sagittal plane, and 640 corresponds to the time points considered (160 × 4, corresponding to 160 Hz and 4 s). Each SMA is formed by the data related to each channel couple combination and is considered independent from the other couples as a separate input pattern. For example, for SMA E, the data related to couple FC1–FC2, couple FC3–FC4, etc., are fed to the network as distinct input patterns during training. The experiment involved the careful selection of channel pairs to target specific brain regions as shown in Figure 2. Henceforth, a dataset refers to a tensor having three dimensions: instances, time points, and channels. The following channel pairs defined areas A through F:

- Area A: [FC1–FC2], [FC3–FC4], [FC5–FC6];
- Area B: [C5–C6], [C3–C4], [C1–C2];
- Area C: [CP1–CP2], [CP3–CP4], [CP5–CP6];
- Area D: [FC3–FC4], [C5–C6], [C3–C4], [C1–C2], [CP3–CP4];
- Area E: [FC1–FC2], [FC3–FC4], [C3–C4], [C1–C2], [CP1–CP2], [CP3–CP4];
- Area F: [FC1–FC2], [FC3–FC4], [FC5–FC6], [C5–C6], [C3–C4], [C1–C2], [CP1–CP2], [CP3–CP4], [CP5–CP6].

### 3.3. Signal Preprocessing

Implementing effective preprocessing methods in EEG-driven motor imagery (MI) systems is critical in elevating data quality and refining classification outcomes. The EEG recordings associated with MI tasks often exhibit artifacts, such as eye blinks and muscle movements, introducing undesired noise. Hence, incorporating indispensable filtering techniques becomes crucial to alleviate these interferences and unveil meaningful neural activity patterns. Moreover, applying signal normalization procedures guarantees uniform and comparable data representation across diverse subjects and sessions, diminishing the impact of individual variations in signal amplitude. Consequently, the designed preprocessing framework integrates both filtering and normalization to cultivate precise and adaptable EEG-based MI signals.

#### 3.3.1. Filtering

The preprocessing included a notch filter at 50 Hz to eliminate power-line interference and enhance signal quality. The EEG data were passed through a fifth-order Butterworth bandpass (BP) filter to preserve the 8–30 Hz frequency range and retain relevant mu rhythm (8~13 Hz) and beta rhythm (14~30 Hz) associated with motor imagery (MI) analysis. This dual-pass filtering method ensures phase integrity while removing low-frequency noise and high-frequency artifacts. Ocular and muscular artifacts have spectral overlap with the underlying EEG and cannot be removed with conventional filtering. Independent component analysis (ICA) is performed to remove artifacts, including eye blinks and muscle activity, maximizing the signal-to-noise ratio and ensuring clean MI-EEG data for accurate analysis and classification.

#### 3.3.2. Normalization

Data normalization is a crucial step in preprocessing EEG signals for motor imagery (MI) brain–computer interface (BCI) systems. This module aims to reduce inter-subject variability, ensuring that features such as baseline participant differences do not influence the classifier’s performance. By normalizing within participants, we aimed to preserve the relative differences in EEG patterns specific to each motor imagery task while reducing the effect of participant-specific global trends or amplitude differences.

The procedure involves the transformation of raw electroencephalography (EEG) signals into a standardized range, typically confined between 0 and 1, leveraging statistical metrics such as mean and standard deviation. This normalization methodology serves to rectify potential biases stemming from variations in amplitude and range across EEG signals recorded from distinct subjects. Additionally, it attenuates the impact of outliers, preventing extreme values from disproportionately influencing the learning process. The selection of a suitable normalization technique is of paramount importance. In this study, z-score normalization is implemented due to its efficacy in preserving the original data distribution while centering it around zero with a standard deviation of one. The ramifications of data normalization in BCI MI systems are profound, leading to enhanced model convergence, improved generalization across heterogeneous datasets, and heightened classification accuracy. Furthermore, normalized data empowers the model to capture pertinent temporal patterns and spatial features within EEG signals, optimizing decoding motor imagery tasks. The z-score used for this purpose is described in Equation (1) as:

(1) $$z=\frac{x-\mu }{\sigma }$$

where ***z*** is the standardized score, ***x*** is the original raw score, ***μ*** is the mean of the dataset and ***σ*** is the standard deviation of the dataset. This formula calculates the number of standard deviations a data point ***x*** is from the mean ***μ***, providing a normalized z-score. Applying this transformation ensures that the data distribution has a mean of 0 and a standard deviation of 1, facilitating comparisons and analyses across different datasets or features.

#### 3.3.3. SMOTE for Data Balancing

Synthetic minority over-sampling technique (SMOTE) data augmentation is employed to counteract class imbalance by generating synthetic samples for underrepresented LF, RF, LRF, and BF, considered as ‘minority classes’. The baseline class, which has the largest number of instances and represents the ‘majority class’, does not undergo this process. The purpose is to increase the instance count of minority classes to match that of the majority class. Synthetic data generation utilizes a k-nearest neighbors algorithm combined with linear interpolation [42]. The technique creates synthetic instances that are interpolations of existing minority class instances. We set the value of k to 5 in our case. Here is a brief overview of how SMOTE operates:

- Selection of Instances:
  - Let $C_{m}$ represent the minority class, and $C_{M}$ represent the majority class.
  - Randomly select an instance $x_{i}$ from $C_{m}$
- Nearest Neighbors:
  - Identify the k-nearest neighbors of $x_{i}$ within $C_{m}$.
- Synthetic Instance Generation:
  - For each neighbor $x_{j},$ calculate the feature difference vector $d_{ij}$ as in (2):
    (2) $$d_{ij}=x_{j}-x_{i}$$
  - Generate a synthetic instance $x_{synth}$ as an interpolation:
    (3) $$x_{synth}=x_{i}+\lambda .d_{ij}$$
    where λ is a random value between 0 and 1.
- Repeating Process:
  - Repeat steps 1–3 until the desired balance between classes is achieved.

New synthetic instances are generated along the line segments between existing minority class samples, balancing all minority classes to the size of the majority class. The validation and test datasets remain unchanged to guarantee that testing and validation rely solely on real data, not synthetic samples.

### 3.4. GRU and Bidirectional (Bi-GRU) Models

**Figure 3:**
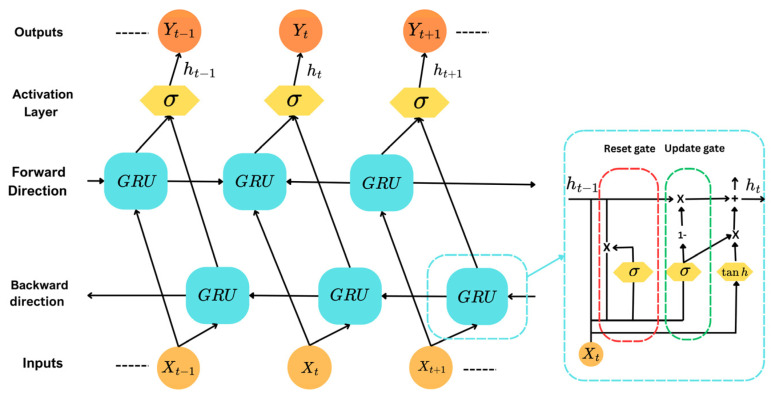
Bi-GRU and GRU architectures. A GRU cell processes input $x_{t}$ and the previous hidden state $h_{t-1}$ using reset and update gates to produce the current hidden state $h_{t}$, capturing temporal dependencies. A Bi-GRU cell employs parallel forward and backward GRU cells, whose outputs are concatenated to integrate past and future context for enhanced temporal pattern decoding.

The proposed MI-BCI system combines a shallow convolutional neural network (CNN) with a gated recurrent unit (GRU) and bidirectional GRU (Bi-GRU). The GRU branch is utilized to enrich temporal representation and boost the classification performance of our hybrid model. GRUs function through reset and update gates, managing past information’s retention and incorporation is depicted in Figure 3. $x_{t}$ represents the input at time step t to the GRU layer and $h_{t}$ denotes the output vector comprising the individual activations $h_{t}^{i}$, where i indexes the GRU units. The output activation of each unit is computed as a linear interpolation between the activation from the previous step $h_{t-1}^{i}$ and a candidate hidden state ${\hat{h}}_{t}^{i}$ controlled by the update gate $z_{t}^{i}$ described in Equation (4):

(4) $$h_{t}^{i}=\left(1-z_{t}^{i}\;\right)\;h_{t-1}^{i}+z_{t}^{i}{\hat{h}}_{t}^{i}$$

The update gate $z_{t}^{i}$ determines the balance between retaining past information and incorporating new information, computed as in Equation (5):

(5) $$z_{t}^{i}=\sigma \left(W_{z}^{i}x_{t}+U_{z}^{i}h_{t-1}+b_{z}^{i}\right)$$

The candidate ${\hat{h}}_{t}^{i}\;$ incorporates the input and modified version of the previous hidden state, modulated by the rest gate $r_{t}^{i}$:

(6) $${\hat{h}}_{t}^{i}=\tanh\left(x_{t}+W_{h}^{i}\;x_{t}+U_{h}^{i}\left(r_{t}^{i}⊙h_{t-1}\right)+b_{h}^{i}\right)$$

where the reset gate $r_{t}^{i}$ is given by:

(7) $$r_{t}^{i}=\sigma \left(W_{r}^{i}x_{t}+U_{r}^{i}h_{t-1}+b_{r}^{i}\right)$$

here W, U, and b are weight matrices and bias of the network. σ() is the sigmoid activation function and ⨀ denotes element-wise multiplication.

### 3.5. Implemented Architectures

The proposed architecture employs a 1D-CNN where convolutional kernels slide over the input pattern along the time dimension. The input matrix has dimensions M×N, with M = 640 (time window length) and N = 2 (two symmetrical EEG channels). The first convolutional layer (conv1) applies 32 kernels of size 20 with a stride of 1 and ’SAME’ padding, ensuring the output size remains unchanged. The second layer (conv2) also uses 32 kernels of size 20 but with ’VALID’ padding. Batch normalization follows conv1 and conv2 to optimize input scaling and accelerate training. A spatial dropout layer with a 50% dropout rate control overfitting by randomly deactivating features.

A third convolutional layer (conv3) with smaller kernels (size 6, ’VALID’ padding) is followed by a 1D average pooling layer (size 2 × 1, stride 2), which reduces the input size, computational cost, and network parameters while improving spatial invariance to small translations. A fourth convolutional layer (conv4), combined with another spatial dropout layer, completes the convolutional sequence. All convolutional layers employ ‘ReLU’ activation function to output the convolution results.

**Figure 4:**
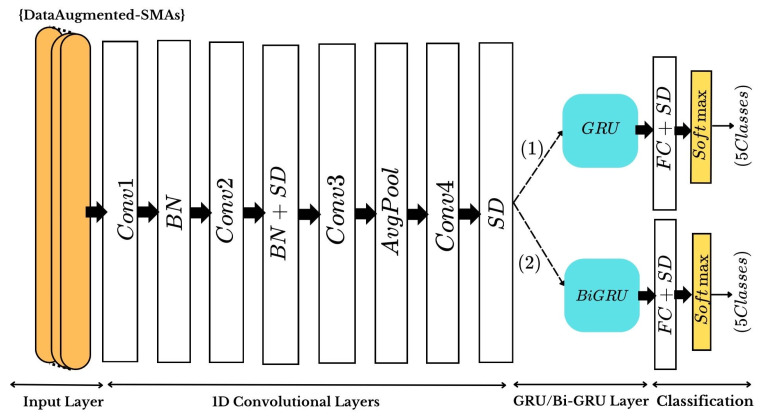
The proposed 1D CNN-GRU/Bi-GRU models architecture: scaled dimensions for a 4 s extracted epoch, using the data augmented of the six sensorimotor channel areas (SMAs) as input, and generating 5 classes (LF, RF, LRF, FF, BF) outputs. BN represents batch normalization, SD refers to the spatial dropout, AvgPool is the average pooling, and FC stands for fully connected layer.

The extracted features are then fed into two branches: (Path1) a GRU layer with 128 units, and (Path2) a Bi-GRU layer with 16 GRU cells of the same size operating sequentially as shown in Figure 4. Finally, the fully connected layers use the ‘Softmax’ activation function to the prediction. The proposed methods were implemented using Python 3.8 frameworks Keras and TensorFlow, with CUDA 11.8 and CUDNN 8.9.7 for acceleration. The computations were performed on an NVIDIA GeForce GTX 1070 GPU (NVIDIA Corporation, Santa Clara, CA, USA). The layer-wise configuration details of CNN-GRU architecture are reported in Table 2.

### 3.6. The Performance Metrics

The performance of the 1D-CNN-GRU and CNN-Bi-GRU models across the six sensory motor areas (SMAs) for each task was evaluated using several performance metrics to provide a comprehensive assessment. The confusion matrix provides a detailed breakdown of the model’s predictions, summarizing the counts of true positives (TP), false positives (FP), true negatives (TN), and false negatives (FN). The elements of the confusion matrix are used to calculate key metrics.

○ The confusion matrix is defined as:

Actual PositiveActual NegativePredicted PositiveTPFPPredicted NegativeFNTN

The following four metrics are derived from the above stated TP, TN, FP, and FN.

○ Accuracy provides an overall measure of correct predictions across all classes:

(8) $$Accuracy=\frac{TP+TN}{TP+TN+FP+FN}$$

○ Precision quantifies the accuracy of positive predictions:

(9) $$Precision=\frac{TP}{TP+FP}$$

○ Recall measures the model’s ability to identify all relevant positive instances.

(10) $$Recall=\frac{TP}{TP+FN}$$

○ F1-Score balances precision and recall, offering a single metric for evaluation:

(11) $$F1-score=\frac{2\times Recall\times Precision}{Recall+Precision}$$

○ Finally, Loss function quantifies the error between predicted probabilities and true labels is defined in Equation (12):
  (12) $$Loss=-\frac{1}{n}\sum_{n}^{i=1}\sum_{j=1}^{m}{\hat{y}}_{ij}{logy}_{ij}$$
  where n is the number of samples, m is the number of EEG classes, $y_{ij}$ represents the predicted output for sample i in class j, and ${\hat{y}}_{ij}$ is the corresponding target value.

## 4. Results

### Classification Results

The classification performance of the CNN-GRU architectures across six selected sensorimotor areas (SMAs) is summarized in Table 3. The table reported the performance metrics computed on the test set averaged overall classes. CNN-GRU achieved accuracies ranging from 96.45% (SMA B) to 99.65% (SMA E), while CNN-Bi-GRU showed a similar trend, with values between 96.66% (SMA B) and 99.64% (SMA E). Notably, SMA E yielded the highest accuracy for both models. These results underscore the nuanced performance variations across different SMAs and reveal the efficacy of the employed architectures in recognizing motor imagery tasks.

**Figure 5:**
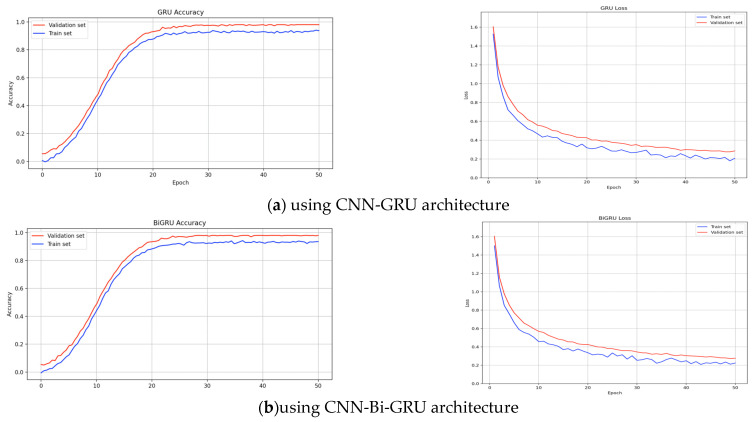
Dynamic changes in loss and accuracy for optimal SMA E throughout training epochs, assessed on training and validation sets.

**Figure 6:**
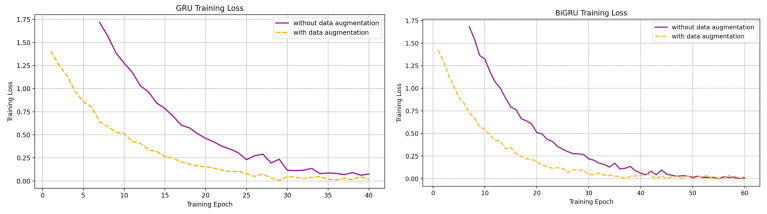
Comparative loss function optimization for CNN-GRU and CNN-Bi-GRU architectures with and without data augmentation.

Figure 5 shows the evolution of loss and accuracy for the best SMA E using training and validation sets. Both CNN-GRU and CNN-Bi-GRU demonstrated a steady loss reduction and improvement in accuracy. Figure 6 compares loss optimization with and without data augmentation, showing stabilization after 30 epochs. The plateau observed in validation curves indicates reduced overfitting. SMOTE data augmentation contributes to faster convergence and stability for both models.

**Figure 7:**
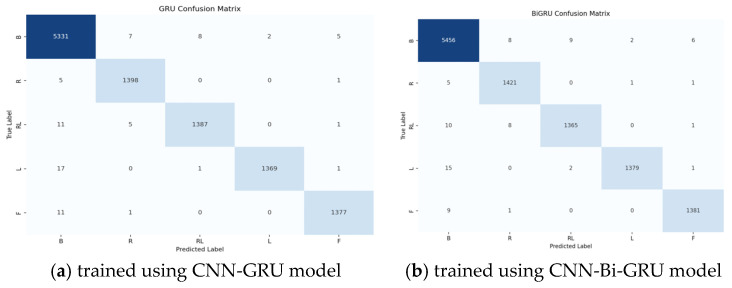
The confusion matrices of LF, RF, LRF, FF, and B tasks classification performance for SMA E on the test set.

Evaluation using additional metrics, such as precision, sensitivity, and F1-score, further confirmed the robustness of the proposed networks. CNN-GRU performance results exceeded 99% for LF, RF, LRF, and BF (Table 4). Both models demonstrated rapid convergence, with CNN-GRU achieving 98.88% accuracy in 36 iterations and CNN-Bi-GRU reaching 98.97% in 38 iterations. High accuracy at the early training phase suggests efficient learning, faster convergence, and resource optimization. Although CNN-Bi-GRU required slightly more iterations, it also exhibited stable convergence, reinforcing its effectiveness. Figure 7 presents confusion matrices for SMA E across five test sets, illustrating the distribution of predicted versus true class labels. The values along the diagonal represent the proportion of accurately classified instances, while the remaining figures denote instances of misclassification. The confusion matrices for both CNN-GRU and CNN-Bi-GRU models outline a strong separation of test data, particularly evident in the best SMA (E), as reflected by high diagonal values. The predominant clustering along the diagonal indicates accurate classifications for all five classes, consistent with the impressive accuracies surpassing 99% reported in Table 4. The baseline class reported strong performance by effectively identifying a high percentage of positive instances. Some instances of misclassification may stem from subjects inadvertently generating motor imagery signals during the baseline state, as individual variations in the timing of motor imagery events can lead to overlapping neural patterns.

**Figure 8:**
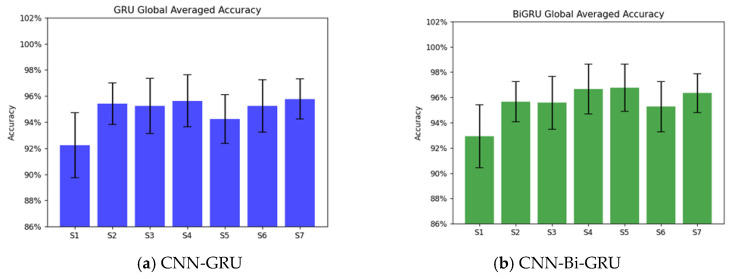
Global averaged accuracy for five motor imagery tasks across seven subjects with selected channels of SMA E. Subfigures (**a**,**b**) depict the individual subject’s global averaged accuracy using column charts for CNN-GRU and CNN-Bi-GRU, respectively.

The individual subject classification was evaluated using seven datasets, testing global average accuracy across motor imagery tasks (LF, RF, LRF, BF, and B). Each subject’s dataset underwent 10-fold cross-validation, dividing data into nine training parts and one testing part, ensuring no data blocks were split. The models underwent training and testing for ten cycles per subject. Figure 8 illustrates the global averaged accuracy for the seven individual subjects for both CNN-GRU and CNN-Bi-GRU models. CNN-Bi-GRU recorded a higher global average accuracy of 96.39% compared to 96.14% for CNN-GRU. Subject 4 achieved the highest accuracy, particularly for left/right fist imaginary movement, while Subject 7 maintained strong consistency with minimal variation. The comparison between the two models revealed that CNN-Bi-GRU attained marginally higher global average accuracy. Both approaches displayed robust and consistent performance across subjects, with minor variations in specific cases (Table 5).

## 5. Comparison and Discussion

The results section demonstrated that our hybrid models successfully classify five distinct motor imagery tasks, demonstrating its ability to diverse neural patterns discrimination. A key contribution of this work is the identification of the most effective sensorimotor area, SMA E, which consists of 12 optimally selected electrodes. This configuration yields the highest classification accuracy, exceeding 99%, highlighting the impact of precise channel selection (Table 3). Meanwhile, integrating SMOTE data augmentation enhances class balance and stabilizes training, leading to faster convergence and reduced overfitting. These findings position our approach as a highly effective and promising solution for EEG-based brain–computer interface (BCI) systems.

Table 6 presents a detailed comparison of methodologies used in various studies, highlighting the number of channels, the number of predicted tasks, and the achieved accuracy. The analysis focuses on works conducted using the same dataset, aligning with our objectives of classifying motor imagery tasks. These studies utilized similar or fewer classification tasks and varying channel configurations. This analysis seeks to clarify how EEG channel selection and network designs impact classification performance.

For instance, our models significantly outperform the method proposed by Hou et al. (2020), which integrates scout EEG source imaging (ESI) with convolutional neural networks (CNN) to classify four motor imagery (MI) tasks without using data augmentation. The method emphasized selecting a region of interest (ROI) using 18 channels within the motor cortex, improved feature extraction, and reported a classification performance of 94.50% [43]. Dose et al. (2018) applied an end-to-end CNN to filter temporal and spatial features and classify four motor imagery tasks. However, the absence of data augmentation and optimized channel selection resulted in extended training times and a lower accuracy of 80.10%, limiting the model’s generalization across different MI tasks [29]. Pinheiro et al. (2018) developed a brain–computer interface for smart wheelchair control, utilizing a single prefrontal cortex EEG channel and extracting FFT-based feature vectors. They employed an artificial neural network (ANN) classifier for a four-class classification task, excluding a baseline class, and achieved a maximum accuracy of 74.96%. While their approach demonstrates the feasibility of EEG-based wheelchair control with minimal channel usage, its moderate classification performance highlights the limitations of a single-channel setup [44].

Karácsony et al. (2019) utilized a CNN classifier to recognize three motor imagery tasks using 16 channels setup with 0.5 s trial inputs and reported an accuracy of 78.6%. The relatively lower accuracy observed with such short trial input can likely be attributed to the lack of data augmentation and the constrained temporal features available for accurate prediction [45].

Lun et al. (2020) obtained a global accuracy of 95.76% using five-layer CNN with two frontal cortex channels. Although this represents the second-best performance after our approach, it involved only four tasks, making it simpler than the five-task classification challenge addressed in our study. The performance gap, with our CNN-Bi-CRU 98.97%, can be explained by the fact that they did not use any form of data augmentation and that the authors restricted their analysis to the (FC3, FC4) channel pair, a subset of the broader sensorimotor areas (SMAs) explored in our study. The reliance on a single channel pair in [31] probably restricted the model’s ability to capture subtle patterns in EEG signals, reducing its effectiveness in more complex scenarios and reinforcing the advantage of our optimized channel selection strategy in enhancing MI task classification. Furthermore, while Lun et al.’s model required 500 iterations to reach stability, our model converged in just 38 iterations, reflecting greater efficiency in training due to the nature of the used 1D CNN strategy.

Li et al. introduced a novel approach to enhance BCI performance in low-data scenarios using the PhysioNet EEG Motor Imagery dataset. Their system incorporated meta-training, which accelerated model convergence by a factor of ten, improving classification accuracy of 80.6% with minimal training data. The system focused only on two motor cortex tasks, using 17 EEG channels configuration [46].

The integration of a hybrid CNN-GRU model with SMA-based channel selection provides two key advantages: (a) It enhances feature extraction by capturing both spatial and temporal dependencies in EEG signals; and (b) it optimizes data representation by selecting the most relevant channel pairs, reducing redundancy and increasing model robustness in motor imagery tasks. Despite its advantages, data augmentation can face limitations when applied to large sensory motor areas (SMA), such as SMA F. As the SMA size increases, it incorporates channels further away from the relevant cortical signal source, risking the introduction of spatial discrepancies that may muddle the network’s performance. In contrast, a smaller SMA, like SMA E, comprises spatially contiguous channels, allowing for meaningful augmentation without excessive distortions due to the electrodes localized positioning.

**Table 6:** Comparison of GRU/CNN and CNN/Bi-GRU performance and the related studies with Physionet dataset.

Models	# Channels	# Classes	Accuracy (%)	Methodology
Dose et al. [29]	64	4	80.10%	CNN
Pinheiro et al. [44]	1	4	74.96%	RNA
Lun et al. [31]	2	4	95.76%	CNN
Hou et al. [43]	18	4	94.50%	ESI-CNN
Li et al. [46]	17	2	80.60%	MAML
Karácsony et al. [45]	16	3	78.60%	CNN
Our works	12	4 + 1	98.88%	CNN/GRU
	12	4 + 1	98.97%	CNN/Bi-GRU

## 6. Conclusions

This study presents a hybrid CNN-GRU model for classifying EEG motor imagery, combining the computational efficiency of a shallow CNN with the temporal dependency handling of GRU modules. The models employed SMOTE data augmentation to balance the dataset, enhancing generalization across all five classes (LF, RF, LRF, FF, and B). Using the Physionet dataset, the model was trained and tested on seven subjects, achieving a macro average accuracy of 98.88% and 98.93% for CNN-GRU and CNN-Bi-GRU, respectively. The highest average accuracy was obtained using the SMA E comprising channel pairs [FC1–FC2], [FC3–FC4], [C3–C4], [C1–C2], [CP1–CP2], and [CP3–CP4], which together encompass the most efficient features from only six channel pairs to enhance BCI MI recognition. With approximately 50 million FLOPs and an inference time of 10 milliseconds in our hardware environment, the models ensure processing pipeline efficiency, particularly with limited computational resources.

Future work will address the challenge of continuous data flow in real-time EEG recordings by implementing dynamic windowing and appropriate transfer learning strategies. We will further validate the method using a self-built database, paving the way for cost-effective, reliable EEG-based BCIs. The presented results reach the uppermost accuracy on group-level classification alongside the use of few discriminative channels within the sensorimotor area, showing the potential of the proposed approach for future development of cheap and practical EEG BCI systems.

## Figures and Tables

**Table 1:** The five motor imagery class labels.

Class Label	Class Description
LF	MI of opening and closing the left fist
RF	MI of opening and closing the right fist
LRF	MI of opening and closing both fists
BF	MI of opening and closing both feet
B	Baseline

**Table 2:** One-dimensional CNN-GRU network architecture details.

Layer Type	Layer Name	Hyperparameters
1st Conv1D Layer	conv1	Filters: 32, Kernel Size: 20, Stride: 1, Padding: ’SAME’, Activation: ReLU
Batch Normalization	BN	Applied to the output of conv1
2nd Conv1D Layer	conv2	Filters: 32, Kernel Size: 20, Stride: 1, Padding: ’VALID’, Activation: ReLU
Batch Normalization	BN	Applied to the output of conv2
Spatial Dropout	SD	Dropout Rate: 50%
3rd Conv1D Layer	conv3	Filters: 32, Kernel Size: 6, Padding: ’VALID’, Activation: ReLU
Average Pooling Layer	AvgPool	Pool Size: 2, Stride: 2
4th Conv1D Layer	conv4	Filters: 32, Kernel Size: 6, Padding: ’VALID’, Activation: ReLU
Spatial Dropout	SD	Dropout Rate: 50%
GRU Layer	gru_1	GRU Units: 128

**Table 3:** Performance analysis of CNN-GRU and CNN-Bi-GRU architectures across the six sensorimotor areas (SMAs) identified in terms of average recall, average precision, average F1, and average accuracy.

Metrics/SMAs	CNN/GRU						CNN/Bi-GRU					
	A	B	C	D	E	F	A	B	C	D	E	F
Average Recall (%)	96.34	95.12	96.23	98.87	**99.33**	98.58	96.46	95.33	96.52	98.91	**99.36**	98.66
Average Precision (%)	97.77	97.17	97.31	99.21	**99.48**	99.13	97.89	97.22	97.41	99.30	**99.52**	99.18
AverageF1-score (%)	97.21	96.19	97.01	99.14	**99.23**	99.11	97.33	96.31	97.23	99.25	**99.44**	99.18
Average Accuracy (%)	96.78	96.45	97.56	99.12	**99.65**	99.12	97.02	96.66	97.78	99.24	**99.64**	99.23

**Table 4:** Results of precision, sensitivity (recall), F1-score, and accuracy, number of training epochs for the proposed CNN-GRU and CNN-Bi-GRU architectures over each class.

Proposed Architectures	Class Label	Precision	Sensitivity	F1-Score	Macro Av. Accuracy	Weighted Av. Accuracy	No. of Training Epochs
CNN/GRU	LF	99.67	99.58	99.13	99.46	99.58	35
	RF	99.85	99.32	99.25	99.77	99.83	32
	LRF	99.33	99.45	99.65	99.27	99.46	41
	BF	99.12	99.47	99.81	99.50	99.54	36
	B	99.28	99.07	99.19	99.46	99.38	33
	Overall	98.25	98.48	98.72	98.88	98.69	36
CNN/Bi-GRU	LF	99.23	99.61	99.69	99.69	99.71	37
	RF	99.73	99.84	99.87	99.87	99.73	42
	LRF	99.66	99.80	99.91	99.39	99.61	33
	BF	99.47	99.61	99.32	99.47	99.86	38
	B	99.48	99.65	99.44	99.44	99.68	37
	Overall	98.47	98.68	98.89	98.97	98.85	38

**Table 5:** Classification results for each subject’s data with corresponding best accuracy task label.

Subject	CNN-GRU		CNN-Bi-GRU	
	Max. Acc. (%) ± Std	Task Label	Max. Acc. (%) ± Std	Task Label
S1	93.87 ± 2.59	LF	93.92 ± 2.43	LF
S2	96.28 ± 1.67	RF	96.67 ± 1.89	RF
S3	96.19 ± 2.12	LRF	96.58 ± 2.32	LRF
S4	97.33 ± 1.98	LRF	97.66 ± 1.99	LRF
S5	95.76 ± 1.87	LF	96.13 ± 1.75	LF
S6	96.27 ± 2.02	BF	96.35 ± 2.11	BF
S7	97.34 + 1.54	B	97.42 + 1.68	B
Global Avg. Acc. (%)	96.14		96.39	

## Data Availability

The data that support the findings of this study are openly available at the following URL/DOI: https://physionet.org/content/eegmmidb/1.0.0/ (accessed on 25 April 2023).
